# Re: Development and validation of MRI-based radiomics model for clinical symptom stratification of extrinsic adenomyosis

**DOI:** 10.1080/07853890.2026.2617748

**Published:** 2026-01-20

**Authors:** Yong Li

**Affiliations:** The First Affiliated Hospital of Hebei North University, Zhangjiakou, Hebei Province, China

Dear editor

Sun et al.’s study presents a valuable attempt to apply MRI-based radiomics to the stratification of symptom heterogeneity in extrinsic adenomyosis, addressing a clinically relevant and underexplored question [1]. The integration of quantitative imaging with clinical variables is technically sound and yields encouraging discriminative performance. To further strengthen the potential of this framework, several design-related considerations deserve discussion.

A key conceptual issue arises from the manner in which clinical symptoms are modeled. Pain, abnormal uterine bleeding (AUB), infertility, and asymptomatic status are treated as independent binary outcomes, while individual patients may contribute to multiple positive symptom categories. Although this reflects real-world clinical presentation, it implicitly assumes symptom independence. Given the well-established overlap and shared pathophysiology among these symptoms in adenomyosis, radiomic features identified as symptom-specific may partly encode global disease severity rather than distinct biological phenotypes. Modeling symptom interdependence through multi-label or hierarchical approaches could therefore improve both specificity and interpretability.

Closely related to biological specificity is the influence of hormonal status at the time of imaging. MRI T2-weighted signal characteristics of the junctional zone and myometrium are known to vary across the menstrual cycle. Radiomic texture features are particularly sensitive to such signal fluctuations. Without accounting for menstrual cycle phase, some associations between radiomics and symptoms may reflect transient hormonal effects rather than stable disease-related imaging traits. Incorporating cycle phase as a covariate or performing targeted sensitivity analyses would help disentangle these effects and enhance reproducibility.

In addition, while the study focuses on lesion heterogeneity captured by texture-based radiomics, symptom expression in extrinsic adenomyosis is strongly influenced by lesion burden and anatomical distribution. The absence of normalization or contextualization of radiomic features to lesion volume or spatial extent limits clinical anchoring, as structural disease characteristics have been associated with clinical manifestations on imaging studies. Integrating hybrid features that combine texture with lesion size or proximity to the endometrium may clarify whether radiomics adds predictive value beyond macroscopic disease characteristics and improve clinical interpretability (e.g., lesion burden and structural changes discussed in recent radiology reviews [2]).

Finally, the classification of “asymptomatic” patients warrants a temporal perspective. Adenomyosis is a progressive condition, and asymptomatic status often reflects an earlier disease stage rather than a distinct phenotype. Radiomic features associated with this group may therefore represent early imaging signatures preceding symptom development. Reframing this task toward predicting future symptom onset, ideally through longitudinal validation, would substantially increase clinical relevance [3].

In conclusion, this study provides a strong methodological foundation for radiomics-based symptom stratification in adenomyosis. Addressing symptom interdependence, hormonal imaging confounders, lesion burden context, and disease temporality may further enhance the biological plausibility and clinical applicability of this approach. Looking ahead, recent studies from 2025 suggest that integrating CT with MRI may offer complementary structural and tissue information that could subtly enhance clinical decision-making. With the growing use of AI-based multimodal image fusion, adding CT on the basis of MRI may help refine assessment in selected cases, although further evidence is still needed to determine whether this combined approach consistently improves patient outcomes [4].

## Data Availability

Data sharing is not applicable to this article as no data were created or analysed in this research.
